# Supplementary figures and images for: Molecular and evolutionary determinants for protein interaction within a class II aldolase/Adducin domain
Source: PLoS One. 2025 Nov 10;20(11):e0316787. doi: 10.1371/journal.pone.0316787 (PMC12599920; doi:10.1371/journal.pone.0316787)

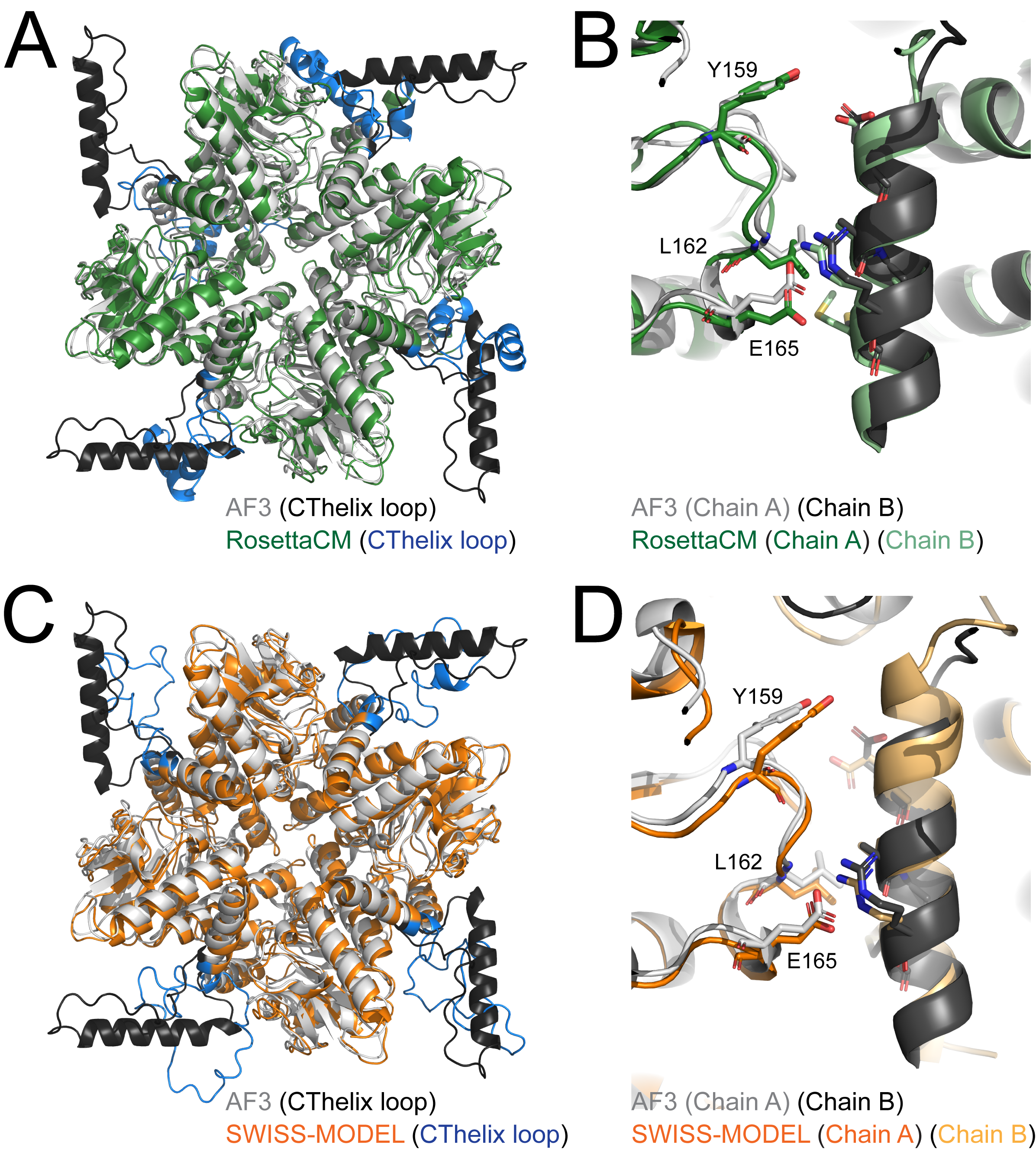

Supplement: S1 Fig — A. Overlay of AF3-generated Hts tetramer (grey core and black CThelices) and RosettaCM homology model (green core and blue CThelices). Strong alignment is seen with all four aldolase domain cores, whereas the CThelices show noticeable divergence. B. Zoom view of chain A-B interface showing similar positioning of the YLE motif, as well as contacting residues in the adjacent CThelix, in AF3 and RosettaCM Hts models. C. Overlay of AF3-generated Hts tetramer (grey core and black CThelices) and SWISS-MODEL homology model (orange core and blue CThelices). Similar to the RosettaCM model, strong alignment is seen with all four aldolase domain cores, whereas the CThelices again display noticeable divergence. D. Zoom view of chain A-B interface showing similar positioning of the YLE motif, as well as contacting residues in the adjacent CThelix, in AF3 and SWISS-MODEL Hts models. (TIF) [file pone.0316787.s001.tif]

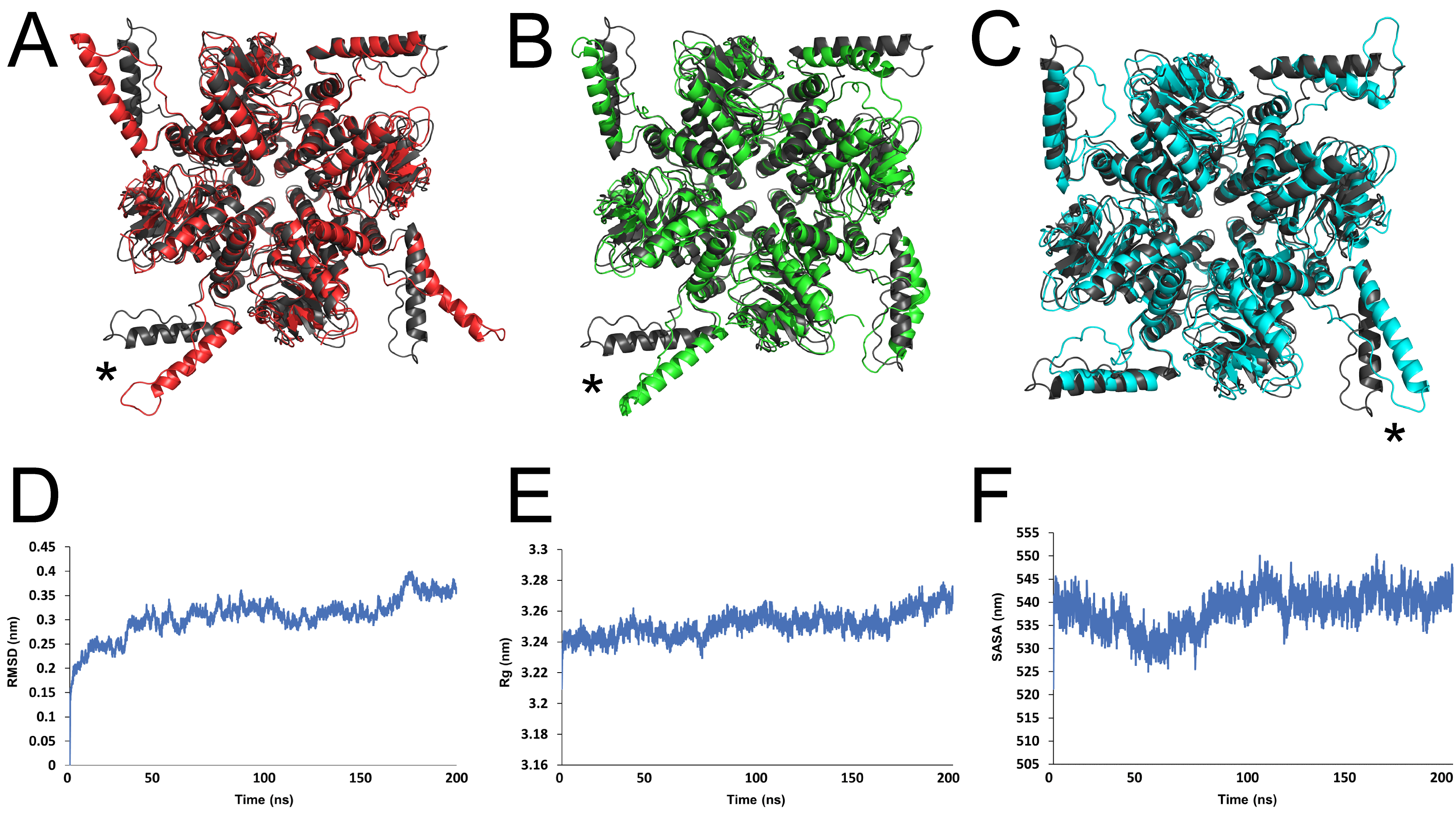

Supplement: S2 Fig — A. Overlay between starting AF3 model (black) and following replicate 1 of a 200 ns MD simulation (red). Asterisk indicates a CThelix showing structural divergence, whereas the core of the tetramer remains stable. B. Overlay between starting AF3 model (black) and following replicate 2 of a 200 ns MD simulation (red). Asterisk indicates a CThelix showing structural divergence, whereas the core of the tetramer remains stable. C. Overlay between starting AF3 model (black) and following replicate 3 of a 200 ns MD simulation (red). Asterisk indicates a CThelix showing structural divergence, whereas the core of the tetramer remains stable. D. Plot of 3 simulation average for RSMD changes throughout 200 ns simulation. E. Plot of 3 simulation average for radius of gyration (Rg) changes throughout 200 ns simulation. F. Plot of 3 simulation average for solvent accessible surface area (SASA) changes throughout 200 ns simulation. (TIF) [file pone.0316787.s002.tif]
