# Supplementary material for: Molecular and evolutionary determinants for protein interaction within a class II aldolase/Adducin domain
Source: PLoS One. 2025 Nov 10;20(11):e0316787. doi: 10.1371/journal.pone.0316787 (PMC12599920; doi:10.1371/journal.pone.0316787)
Supplement: S1 Table — Listed are the gene description, organism, and NCBI URL link for specific protein sequences used to identify related sequences across taxa. (DOCX) [file pone.0316787.s003.docx]

| Gene Description | Organism | NCBI URL Link |
| --- | --- | --- |
| Class I enzyme | *Drosophila melanogaster* | <https://www.ncbi.nlm.nih.gov/protein/NP_001262985.1?report=genbank&log$=protalign&blast_rank=1&RID=M9M6K9CS013> |
| Class II enzyme | *Escherichia coli* | <https://www.ncbi.nlm.nih.gov/protein/EFE61766.1?report=genbank&log$=protalign&blast_rank=2&RID=M9MBFN75016> |
| Class IIa enzyme (‘Full CT’) | *Pseudomonas* | <https://www.ncbi.nlm.nih.gov/protein/WP_085599007.1?report=genbank&log$=protalign&blast_rank=1&RID=M9MNMM3R016> |
| Class IIa enzyme (‘Half CT’) | *Enterobacteriaceae* | <https://www.ncbi.nlm.nih.gov/protein/WP_000440781.1?report=genbank&log$=protalign&blast_rank=1&RID=M9NB2A1X016> |
| Class IIa enzyme (‘No CT’) | *Aquifex aeolicus* | <https://www.ncbi.nlm.nih.gov/protein/WP_010881293.1?report=genbank&log$=protalign&blast_rank=1&RID=M9NJDBMR016> |
| Class IIa/Adducin domain | *Drosophila melanogaster* | <https://www.ncbi.nlm.nih.gov/protein/NP_001246421.1> |

**Table S1: List of sequences used for BLAST-based database searches related to results presented in Figure 9.**
