# Supplementary material for: Molecular and evolutionary determinants for protein interaction within a class II aldolase/Adducin domain
Source: PLoS One. 2025 Nov 10;20(11):e0316787. doi: 10.1371/journal.pone.0316787 (PMC12599920; doi:10.1371/journal.pone.0316787)

Original digital images of gels shown in Figure 2

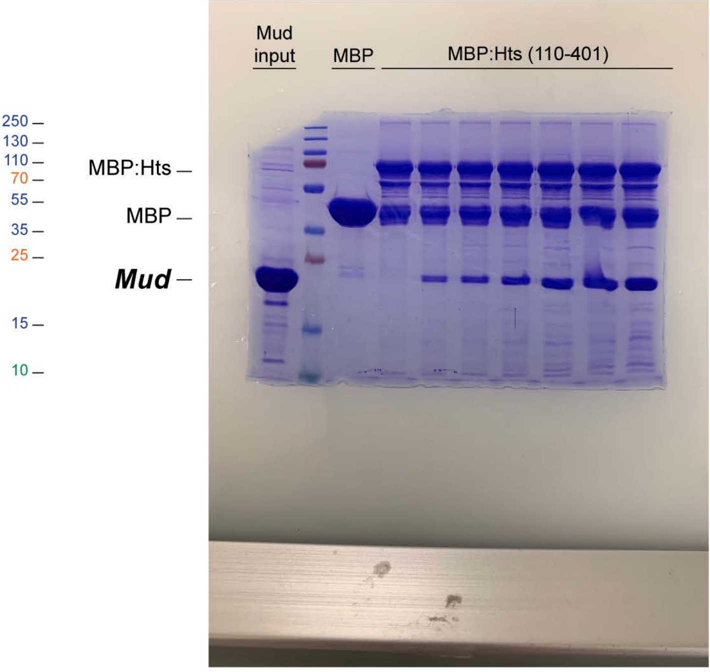

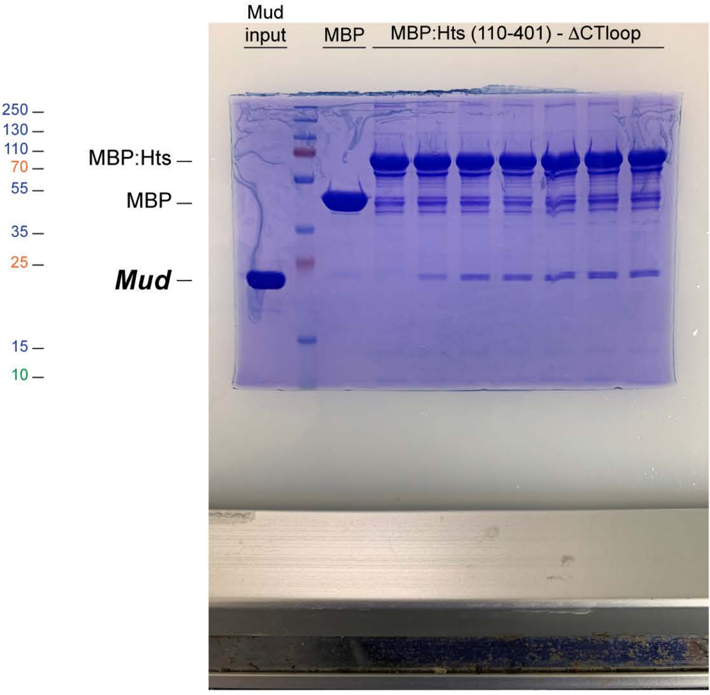

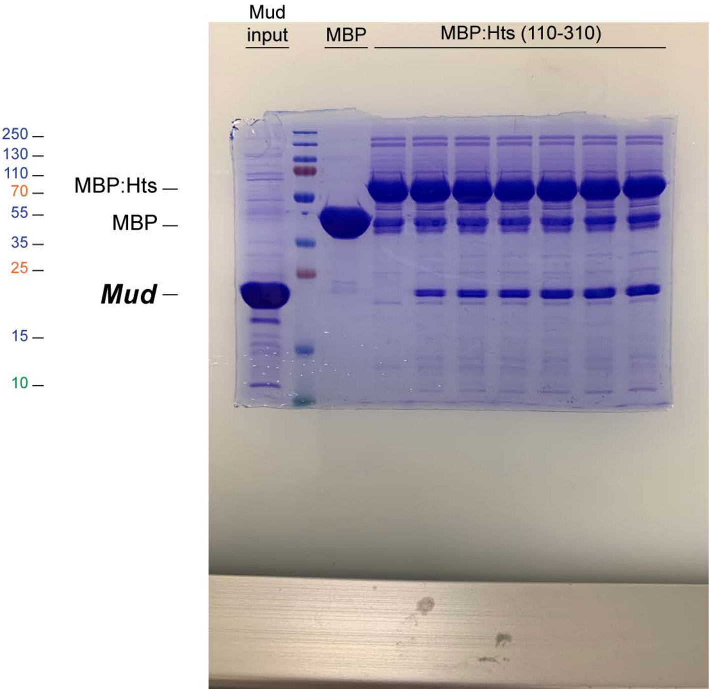

Original digital images of gels shown in Figure 3

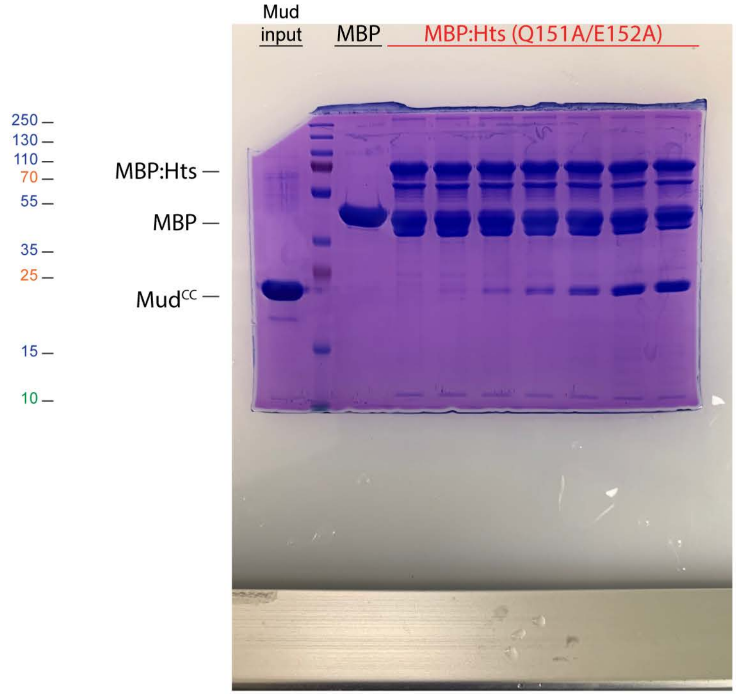

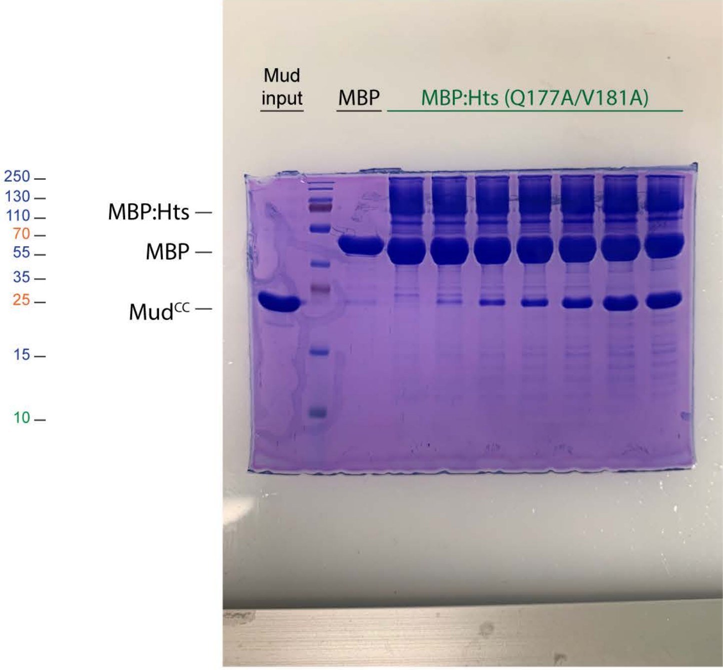

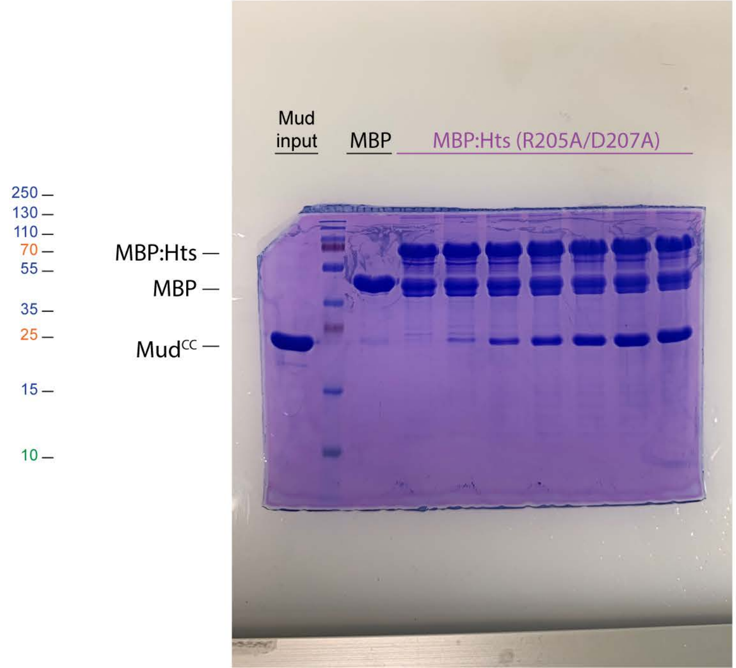

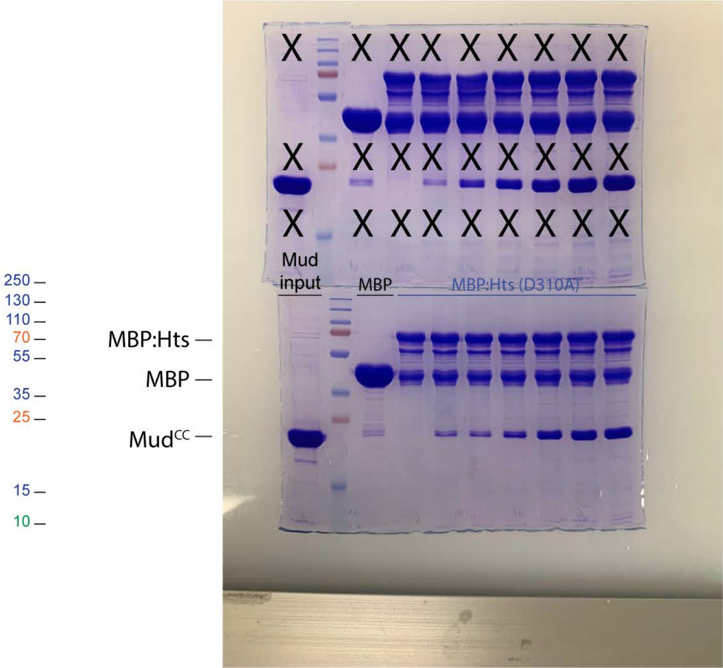

Original digital images of gels shown in Figure 4

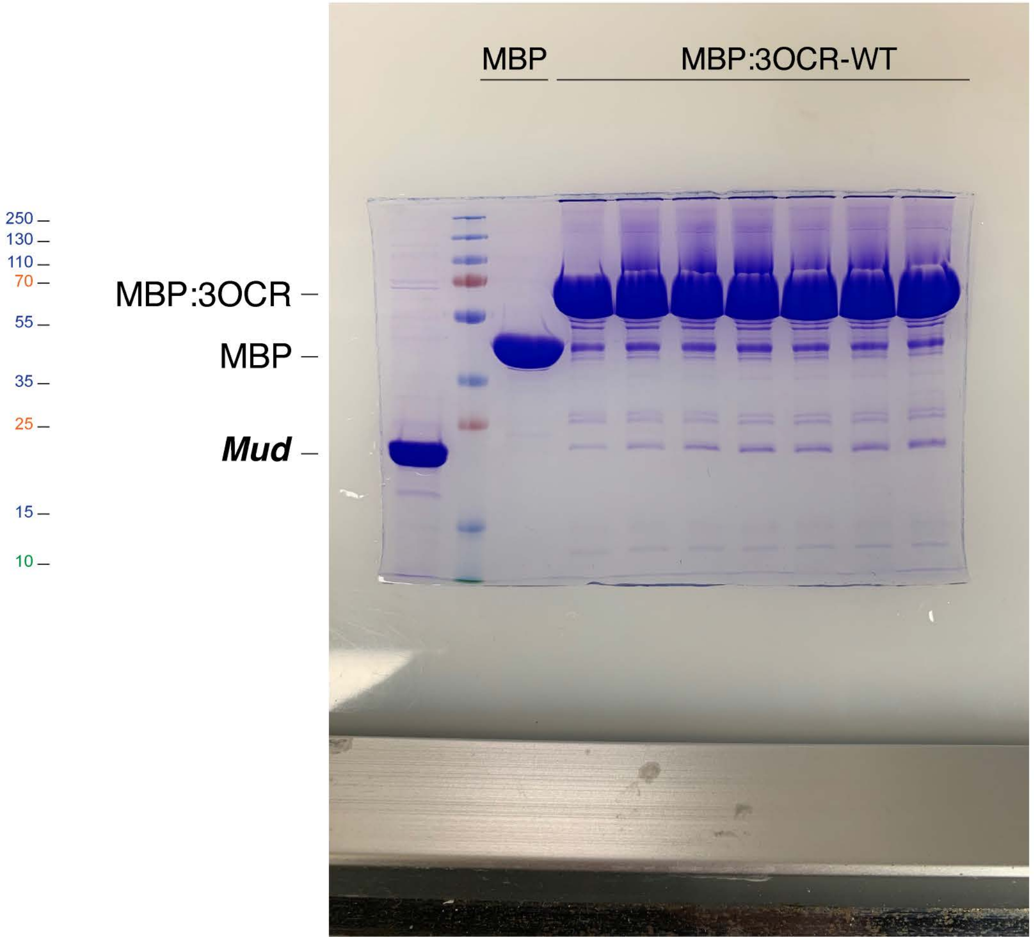

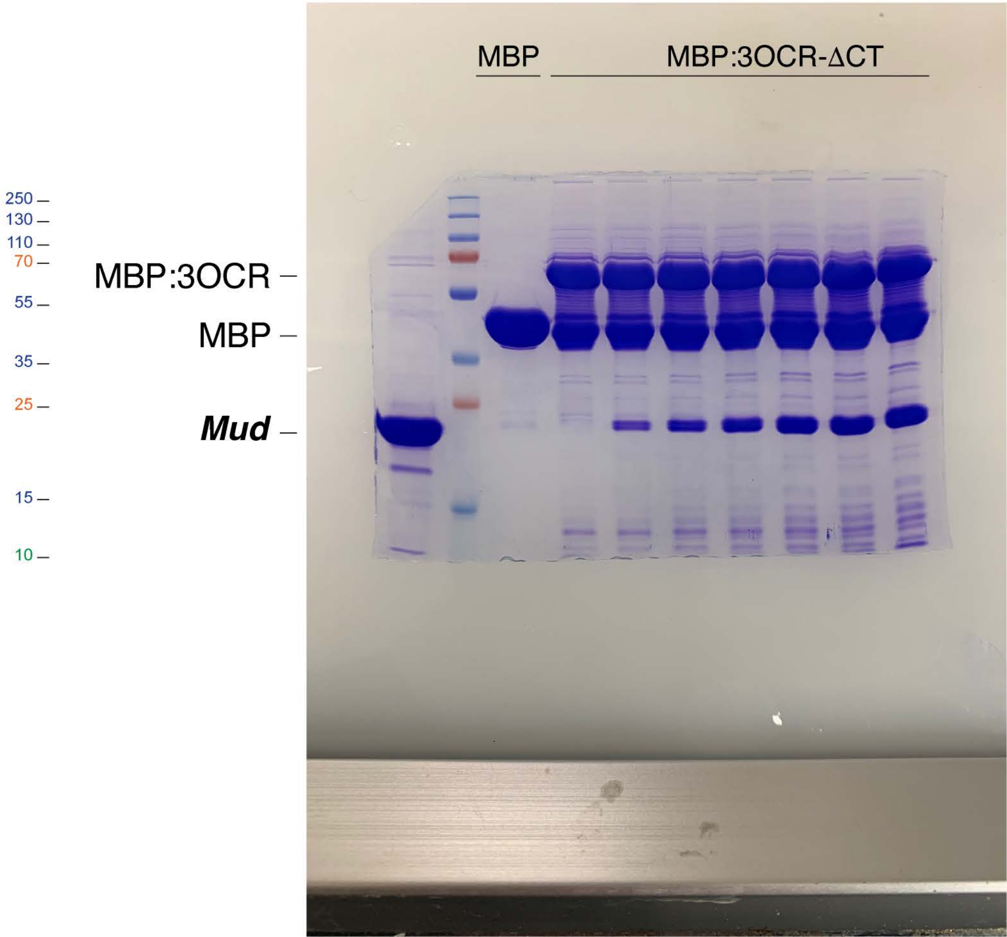

Supplement: S1 File — Compilation of all original digital images for SDS-PAGE gels. These images lack any cropping or adjustments that were made in the preparation of final images presented in relevant Figures. (PDF) [file pone.0316787.s004.pdf]
