# Supplementary material for: Molecular and evolutionary determinants for protein interaction within a class II aldolase/Adducin domain
Source: PLoS One. 2025 Nov 10;20(11):e0316787. doi: 10.1371/journal.pone.0316787 (PMC12599920; doi:10.1371/journal.pone.0316787)
Supplement: Attachment — Submitted filename: PONE-D-24-58129_Response to reviewers.docx [file pone.0316787.s006.docx]

Additional Editor Comments:

The manuscript titled "Molecular and evolutionary determinants for protein interaction within a class II aldolase/Adducin domain" investigates the functional evolution of the class II aldolase domain (ALDODOM), examining its transformation from an enzymatic protein to a protein-binding domain. Using AlphaFold-based modeling, biochemical binding assays, and evolutionary sequence analysis, the study focuses on the interaction between the Drosophila ALDODOM and the spindle regulator, Mud, uncovering critical molecular determinants that facilitated this functional transition. While the research is robust and provides significant insights into protein evolution, improvements in organization, clarity, and presentation could enhance the manuscript’s accessibility and impact. Below are my detailed suggestions for refinement:
Major Points for Improvement
Introduction:
• The introduction provides adequate context but would benefit from greater conciseness and improved structure. A streamlined narrative emphasizing the evolutionary and functional transitions of ALDODOM would enhance reader engagement.

- ***To keep the introduction more focused on Aldolase function and evolution, we have moved the discussion of GK evolution to the discussion, which is now found on Lines 583-605.***

Methodology:
• The materials and methods section is comprehensive but could be better organized. Dividing it into distinct subsections with numbered headers for structural modeling, protein purification, and assays would improve readability.

- ***We have added numberings (1-5) for subsections in the Methodology section for better clarity.***

• Include more explicit details on the statistical analysis for biochemical assays, such as error margins, sample sizes, and replicates, to ensure reproducibility and transparency.

- ***We have added some additional language to help better explain the number of measurements in each experiment and the number of replicates used to generate binding graphs shown in the figures. This can be found on lines 165-167 and 173-174.***

Results:
• The results section is thorough but could be presented more clearly by breaking it into smaller subsections with descriptive subheadings (e.g., "Role of the YLE motif," "Impact of CThelix truncation"). This would make key findings more accessible.

- ***We have broken up several larger sections of the Results and added additional subheadings to make them more accessible to the reader as suggested.***

• Some figure captions are overly technical and could be simplified while retaining necessary details to aid comprehension.

- ***We have modified most figure captions in an attempt to simplify their presentation for readers.***

• Consider reformatting figures with clearer labeling and more distinct color schemes, particularly for structural overlays, to emphasize features discussed in the text.

- ***Many of the structural overlays contain many highlighted segments requiring use of unique colors, some of which are less bright than others unfortunately. We considered alternative color schemes but did not feel they improved visibility.***

Discussion:
• The discussion integrates the findings with broader evolutionary implications effectively but could benefit from highlighting unanswered questions and proposing directions for future research.

- ***We have added an additional paragraph at the end of the Discussion section that offer a few additional future research directions that our study supports.***

• Incorporate recent references on advancements in structural modeling tools and studies on evolutionary transitions in protein domains to provide contemporary context.

- ***We have added short discussions of the following references on protein domain evolution. These have been added to the Introduction and/or Discussion sections of the revised manuscript:***
  - ***B. K. Muralidhara (2008)***
  - ***M. J. Harms (2014)***
  - ***A. F. Dishman (2021)***
  - ***A. Kwon (2019) and J. Boudeau (2006)***
  - ***A.S. Pillai (2022)***
  - ***G.K. Hochberg (2017)***

• Expand the comparative analysis with other evolutionary examples (e.g., guanylate kinase evolution) to draw parallels and contrasts that reinforce the significance of the findings.

- ***We have attempted to expand the discussion of GK evolution and provide a better comparison with the present findings as suggested. This includes a more thorough discussion of both the similarities and distinct differences compared to the current findings presented here with aldolase.***

Figures and Tables:
• Enhance the clarity of structural overlay figures with improved labeling and distinct colors to differentiate discussed features.

- ***As mentioned above, we considered alternative color formats but were unsatisfied with the results and did not feel they improved figure presentation.***

• Add a table summarizing key mutations, their evolutionary significance, and functional impacts to consolidate the findings effectively.

- ***We have added ‘Table 1’ with information suggested. We agree that this improves reader access to key findings from the rather extensive list of mutants presented in the manuscript.***

Major Points for Improvement
• Conduct a thorough review to eliminate grammatical errors, typos, and verbose sections.

- ***We have reviewed the manuscript and attempted to correct all grammatical errors.***

• Simplify overly technical language to improve readability without compromising scientific accuracy.
By addressing these areas, the manuscript can achieve a more refined and impactful presentation, making it more accessible to a broader audience while maintaining its scientific rigor.

- ***We have attempted to improve readability to a broad audience by minimizing technical language as requested.***

**Comments to the Author**


Please use the space provided to explain your answers to the questions above. You may also include additional comments for the author, including concerns about dual publication, research ethics, or publication ethics. (Please upload your review as an attachment if it exceeds 20,000 characters)

Reviewer #1: The manuscript is interesting. However, I have certain issues:
1. The authors used alfa fold for structure generation. They should try other methods also and compare their results. Since the CryoEM structure of a homologous protein is available, the authors must try homology modelling.

- ***We have completed two homology modeling experiments to complement the AlphaFold-3 structure as suggested. This includes a model based on the CryoEM structure of porcine Adducin heterotetramer using Rosetta Comparative Modeling, along with a second homology model using SWISS-MODEL and the homotetrameric aldolase enzyme crystal structure from Pseudomonas. We appreciate the suggesting and believe this adds additional confidence of the Hts modeling and insights into its structural state. Results are found in Lines 243-251, 360-369, and in Supplemental Figure S1.***

2. The built structure must be validated using MD simulations in triplicate.

- ***We include in the revised manuscript MD simulations as requested. These simulations were done in triplicate, with additional details included in the updated Materials and Methods section (see subsection “5. Molecular Dynamics (MD) simulations:” Lines 190-198). These experiments further validate the tetrameric assembly of the Hts aldolase domain and indicate that it is a stable assembly. Results are found in Lines 252-264 and in Supplemental Figure S2.***

3. The binding assay presented here must also be validated by docking simulations using at least 3 different protocols.

- ***We thank the reviewer for this suggestion but have not included docking simulations in our revised manuscript. The binding assays and protocols used are a standard approach that alone represent a quantitative and empirical assessment of Hts/Mud interactions and effects of various mutations on binding. As such, we believe that our interpretations of the results are not contingent on docking experiments. We hope the reviewer respects our decision on this point.***
