# Supplementary material for: Molecular and evolutionary determinants for protein interaction within a class II aldolase/Adducin domain
Source: PLoS One. 2025 Nov 10;20(11):e0316787. doi: 10.1371/journal.pone.0316787 (PMC12599920; doi:10.1371/journal.pone.0316787)
Supplement: Attachment — Submitted filename: PLOS ONE Reviewer Report.docx [file pone.0316787.s007.docx]

**PLOS ONE Reviewer Report**

Manuscript Title: **Molecular and evolutionary determinants for protein interaction within a class II aldolase/Adducin domain**

**Recommendation to the Editor:** This manuscript is suitable for publication with **minor revisions.**

**Comments:**

Lines 34–43: The explanation of neofunctionalization and domain evolution contains redundant wording. Condense this section to enhance readability and focus.

Line 74–77: The sentence “Thus... remains unknown.” is vague. Rephrase it to clearly state the hypothesis being tested.

Lines 186–211: The long list of BLAST URLs in the Methods section is distracting. Consider relocating URLs to the supplementary data or the data availability section.

Lines 215–259: The section on structural modelling is technically dense and difficult to follow. Add a summarizing sentence at the end to explain how the modelling informed or guided hypothesis generation.

Lines 437–454: The *Trichoplax* analysis is strong but densely written. Insert a brief sentence explaining why *the Placozoa* is evolutionarily significant.

Lines 617–621: “through co-option of the enzyme GMP substrate binding site…” is not grammatically incorrect, but it is potentially unclear. Revise it for clarity and grammatical accuracy. When describing domain repurposing or neofunctionalization, clarity is key, especially for enzyme active sites or binding pockets. Therefore, a slight rephrasing will improve reader comprehension without altering the scientific meaning.
