# Supplementary material for: Molecular and evolutionary determinants for protein interaction within a class II aldolase/Adducin domain
Source: PLoS One. 2025 Nov 10;20(11):e0316787. doi: 10.1371/journal.pone.0316787 (PMC12599920; doi:10.1371/journal.pone.0316787)
Supplement: Attachment — Submitted filename: PONE-D-24-58129R2_Response to reviewers.docx [file pone.0316787.s008.docx]

**PLOS ONE Reviewer Report**

Manuscript Title: **Molecular and evolutionary determinants for protein interaction within a class II aldolase/Adducin domain**

**Recommendation to the Editor:** This manuscript is suitable for publication with **minor revisions.**

**We would like to sincerely thank the reviewer for their time and willingness to review our manuscript and offer insightful feedback. We believe we have addressed each of the comments and that our revised manuscript is improved as a result. Below are specific responses to the reviewer’s comments.**

**Comments:**

Lines 34–43: The explanation of neofunctionalization and domain evolution contains redundant wording. Condense this section to enhance readability and focus.

- **We have attempted to reword this section to be more concise and focused by removing redundant wording as suggested. We believe the paragraph has improved through this advice. The revised section is now Lines 33-38.**

Line 74–77: The sentence “Thus... remains unknown.” is vague. Rephrase it to clearly state the hypothesis being tested.

- **We have attempted to rephrase this sentence to be a more clear and direct statement of the study’s goal as suggested. This can be found on Lines 82-85 of the revised manuscript.**

Lines 186–211: The long list of BLAST URLs in the Methods section is distracting. Consider relocating URLs to the supplementary data or the data availability section.

- **We have moved all URL links to a new Supplementary Table S1 as suggested. We agree this makes this section of the methods much less distracting in its presentation. A reference in the main text is located on Lines 197-199.**

Lines 215–259: The section on structural modelling is technically dense and difficult to follow. Add a summarizing sentence at the end to explain how the modelling informed or guided hypothesis generation.

- **We have added two sentences at the end of this paragraph. The first adds additional summary of the key interpretation of the modelling, and the second makes clear that these initial results were an important step to the remainder of the study on how Hts and Mud interaction. These revisions can be found on Lines 250-254.**

Lines 437–454: The *Trichoplax* analysis is strong but densely written. Insert a brief sentence explaining why *the Placozoa* is evolutionarily significant.

- **We have added some additional, short descriptions of Placozoa and *Trichoplax* that provide more perspective on their evolutionary significance, along with additional references for readers. These can be found on Lines 453-456 and 461-462 in the revised manuscript.**

Lines 617–621: “through co-option of the enzyme GMP substrate binding site…” is not grammatically incorrect, but it is potentially unclear. Revise it for clarity and grammatical accuracy. When describing domain repurposing or neofunctionalization, clarity is key, especially for enzyme active sites or binding pockets. Therefore, a slight rephrasing will improve reader comprehension without altering the scientific meaning.

- **We have made a slight modification to this text to hopefully reduce any confusion among readers as suggested. This new phrasing can be found on Lines 629-631 in the revised manuscript.**
